# Outbreak of KPC-2 Carbapenem-resistant *Klebsiella pneumoniae* ST76 and Carbapenem-resistant K2 Hypervirulent *Klebsiella pneumoniae* ST375 strains in Northeast China: molecular and virulent characteristics

**DOI:** 10.1186/s12879-020-05143-y

**Published:** 2020-07-02

**Authors:** Shanshan Su, Jisheng Zhang, Yongxin Zhao, Lan Yu, Yong Wang, Yuchao Wang, Mingjia Bao, Yu Fu, Chunjiang Li, Xiaoli Zhang

**Affiliations:** 1 Yongchuan hospital of Chongqing Medical University, Chongqing, China; 2 The First People’s Hospital of Jingzhou City, Jingzhou, Hubei China; 3 First Affiliated Hospital of Jiamusi University, Jiamusi, Heilongjiang China; 4 Center for Disease Control and Prevention, Jiamusi, Heilongjiang China

**Keywords:** Carbapenem resistance, *Klebsiella pneumoniae*, Virulence, Epidemiology, Whole-genome sequencing

## Abstract

**Background:**

Carbapenem-resistant hypervirulent *Klebsiella pneumoniae* strains have recently come into existence worldwide; however, researchers in northeast China are not aware of their clinical features and molecular characteristics.

**Methods:**

Here, the molecular and virulent characteristics of 44 carbapenem-resistant *K. pneumoniae* (CRKP) isolates collected from January 2015 to December 2017 were studied. Multilocus sequence typing (MLST) and pulsed-field gel electrophoresis (PFGE) were carried out to define the clonal relatedness among the isolates. PCR and capsular serotyping of the virulence-associated genes, as well as biofilm formation and serum complement-mediated killing assays, were employed to determine the virulent potential. The genomic features and associated mobile genetic elements of JmsCRE57 were detected by whole genome sequencing.

**Results:**

The only positive isolate was JmsCRE57, which belonged to the ST375 serotype K2 that expressed *uge*, *mrkD*, *fimH*, *kpn*, *aerobactin* and *rmpA* virulence-associated genes and showed strong biofilm formation and serum sensitivity. Sequencing results showed that the JmsCRE57 genome mainly consisted of a circular chromosome, three antimicrobial resistant plasmids and a virulent plasmid. The antimicrobial resistant plasmid expressing *bla*_*KPC-2*_, *bla*_*CTX-M-15*_, *aph(3″)-Ib*, *aph(6)-Id*, *qnrB1*, *aac(3)-IIa*, *aac(6′)-Ib-cr*, *bla*_*OXA-1*_, *bla*_*TEM-1B*_, *catB4*, *sul2*, *dfrA14* and *bla*_*SHV-99*_. The virulent plasmid belonged to the IncHI1B group, which is mainly composed of mucoid phenotype genes and siderophore-associated genes. The remaining CRKP strains that expressed *uge*, *fimH*, *mrkD* and *kpn* virulence-associated genes were not successfully typed.

**Conclusion:**

Our results provide new insights on the epidemiology of carbapenem-resistant K2 hypervirulent *K. pneumoniae* ST375 and CRKP ST76 strains in northeast China, which may help control their future outbreaks.

## Background

*Klebsiella pneumoniae* has become a common pathogen that is often treated in clinical practices. It normally causes pneumonia, bacteremia, urinary tract infections, and surgical-site infections in hospitalized patients [1]. With the increasing overuse of common antibiotics, carbapenem-resistant *Klebsiella pneumoniae* (CRKP) strains have spread worldwide in the past two decades. CRKP infections increase the length of hospitalization, and their treatment results in higher overall costs. Its mortality rate is as high as 40–50%, which has attracted significant public attention [2].

The hypervirulent *K. pneumoniae* (hvKP) strain was first discovered in Taiwan in 1982 [3], and its presence was later reported by the United States, Australia, Mexico, and South Korea [4, 5]. The hvKP strain gets its name from its ability to cause community-acquired liver abscesses in young, healthy individuals. In addition, it may cause extrahepatic complications, including necrotizing fasciitis, endophthalmitis and meningitis [6]. Presently, there are at least 78 serotypes of *K. pneumoniae* worldwide, and serotypes K1 and K2 can cause liver abscesses [7]. These serotypes are largely characterized by their ability to produce capsular polysaccharides, which is typified by a super-viscous phenotype that enables them to avoid phagocytosis by neutrophils [8]. Several virulence factors, including the genes that regulate the mucoid phenotype A (*rmpA*) and siderophore production (*aerobactin*), have been shown to be major virulence genes of hvKP.

Unlike the multi-drug resistant form of *K. pneumoniae*, the hvKP strain is mostly sensitive to antimicrobials other than ampicillin. With the horizontal transmission of *K. pneumoniae* carbapenemases (KPC), however, New Delhi metallo-beta-lactamase (NDM) and other carbapenemases appeared as carbapenem-resistant hypervirulent *K. pneumoniae* that are highly aggressive and capable of escape from the host’s immunological response [9]. This strain has already caused public panic due to its incurability, and its presence has been reported by major cities such as Taiwan, Beijing, Nanchang and Zhejiang [3, 4, 7, 10]. In addition, its high mortality and prevalence rates call for further studies. We previously reported the mechanism responsible for the resistance of CRKP to antimicrobials [11]. In this study, we address the virulence of CRKP isolates, which will enable us to compare the molecular characteristics and the virulence of the ST76 CRKP strain with the KPC-2 resistance gene in this region and to provide epidemiological data for patients infected with CRKP. To the best of our knowledge, this is the first report to describe the genomic background and the virulence of the carbapenem-resistant K2 hypervirulent *K. pneumoniae* ST375 strain in Heilongjiang Province, Northeast China.

## Methods

### Collection and identification of *K. pneumoniae* isolates

Forty-four CRKP isolates were collected from patients at the 1980-bed First Affiliated Hospital of Jiamusi University in Heilongjiang Province, northeast China, from January 2015 to December 2017. The isolates were identified as CRKP strains by the VITEK-2 System (bioMe’rieux). The minimal inhibitory concentrations (MICs) of imipenem and meropenem were verified by the E-test, and the results were interpreted according to the 2016 Clinical and Laboratory Standard Institute Guidelines. Hypermucoviscosity was determined using the string test. Quality control strains (*Escherichia coli* ATCC 25922, *Salmonella* H9812 and *K. pneumoniae* ATCC 700603) were used for pulsed-field gel electrophoresis, as well as antimicrobial susceptibility and serum complement-mediated killing assays. Nine out of forty-four CRKP strains died after completing the molecular biology experiments due to improper preservation.

### Multilocus sequence typing (MLST) and pulsed-field gel electrophoresis (PFGE)

MLST was used to screen the 44 CRKP strains by amplifying seven housekeeping genes (*gapA*, *infB*, *mdh*, *pgi*, *phoE*, *rpoB* and *tonB*) expressed by *K. pneumoniae* according to the protocol at (http://bigsdb.pasteur.fr/klebsiella/primers_used.html). eBURST Software (*ver* 3) was used to analyze the sequence types (STs). Clonal complexes (CCs) were defined as those originating from the same genotype; they shared alleles with another member of the group at six out of seven loci and predicted the ST with the largest number of a single locus variant (SLV). PFGE was performed on 35 CRKP strains that were digested with *Xba*I for 3 h at 37 °C. The digested fragments were separated on a 1% Seakem Gold agarose gel for 18 h at 14 °C using the Bio-Rad CHEF MAPPER System. The band patterns were analyzed using BioNumerics 7.0 Software. Clusters were defined as DNA patterns sharing ≥85% similarity. PFGE patterns were identified as previously described [12].

### String test

CRKP strains were incubated overnight on blood agar. A single colony was touched with a loop and stretched outward. The length of the viscous string was pulled upward and measured. A positive string test result was defined as a string longer than 5 mm. The string test was repeated three times for each strain, and determined the final result.

### Detection of capsular serotyping and virulence-associated genes

Forty-four CRKP strains belonging to K1, K2, K5, K20, K54 and K57 serotypes were identified by PCR [13]. Virulence-associated genes (*rmpA*, *uge*, *magA*, *kfu*, *mrkD*, *fimH*, *kpn*, *iroNB*, *alls*, *wcaG* and *aerobactin*) were amplified by PCR as previously described [14–16]. The amplified transcripts were sequenced, and BLAST was used to determine their identities.

### Biofilm formation assay

In brief, 10 μl of the 0.5 McFarland bacterial standard and 200 μl of Luria-Bertani (LB) broth were inoculated into the wells of a 96-well microplate, with four wells per strain, and the microplate was incubated at 37 °C for 24 h. Thereafter, the LB broth was removed, and the bacterial cells were stained with 200 μl of 0.1% crystal violet at room temperature for 15 min, then removed the due. The wells were washed free of dye with PBS and then dried. The absorbance was measured with a microplate reader set at 570 nm after adding 200 μl of ethanol for 10 min into the wells. The yield of biofilm formation of the strains was interpreted as follows: OD > 0.6 as strong-producing, 0.4 < OD ≤0.6 as moderate-producing and OD < 0.4 as weak-producing.

### Serum complement-mediated killing assay

Venous blood was collected from 10 healthy volunteers, who had provided written informed consent before participation in the studies. Sera were obtained and stored at − 80 °C until use. A bacterial stock at mid-log-phase was diluted to 1 × 10^6^ colony-forming units (CFUs)/ml in 0.9% saline, combined with serum at a 1:3 volume ratio, and then incubated at 37 °C. Serial dilutions were plated on MHA and incubated for 0, 1, 2, and 3 h to determine the number of colonies. Each sample was tested three times. The results were presented as means, and the final results were expressed as previously described [17]. *K. pneumoniae* ATCC 700603 and *K. pneumoniae* Jms100, which exhibits a hypermucoviscous phenotype and is sensitive to all antimicrobials except ampicillin, was isolated from a liver abscess from a patient in our hospital and used for comparison.

### Whole genome sequencing

JmsCRE57 genomic DNA was extracted from overnight cultures using the QIAamp DNA Mini Kit (Qiagen, Hilden, Germany). The Illumina HiSeq 2000 System (Illumina Inc., San Diego, CA, USA), which generates 300–500 bp paired-end sequences, and the PacBio System (Pacific Biosciences, Menlo Park, CA, USA), which assembles a 10-kb fragment library, were used via SOAPdenovo (*ver* 2.04). The genomic sequences were annotated using the Prokka 1.12 Program. The expression of rRNAs and tRNAs was predicted using Barrnap 0.4.2 Software and tRNAscan-SE Software (*ver* 1.3.1), respectively, whereas bacterial gene expression was predicted using Glimmer 3.02 Software. The annotated information for the predicted genes was obtained using BLAST aligned with NRGene, EggNOG and GO Databases. The PlasmidFinder Database and BLASTn were used to identify the incompatibility groups. The antimicrobial resistance genes and virulence genes were identified after uploading the assembled genome at ResFinder (https://bitbucket.org/genomicepidemiology/resfinder) and the Virulence Factor Database (VFDB) (http://www.mgc.ac.cn/VFs/). The JmsCRE57 genomic sequence was deposited into GenBank under accession number SAMN10995714.

### Statistical analysis

Statistical analysis was performed using IBM SPSS Statistics Software (*ver* 20.0) and Graphpad Prism Software (*ver* 7). Data were presented as medians or means ± standard deviation.

## Results

### Clinical characteristics of CRKP isolates

The clinical characteristics and antimicrobial susceptibility of 44 CRKP isolates obtained from different clinical specimens, including 38 respiratory secretions (86.3%), five blood specimens (13.6%) and one wound (2.3%), were investigated. The mean ± standard deviation age of the patients was 60.3 ± 15.2 (range, 16–86) years. Most CRKP isolates were obtained from patients at the neurology unit and ICU, with a separation rate of 40.9 and 38.6%, respectively, followed by 9.1, 6.8, 2.35 and 2.35% at the units of emergency, hematology, orthopedics and cardiac surgery, respectively. The mortality rate was 27.3%. Most patients presented with severe underlying diseases and received several antimicrobials during hospitalization. The clinical characteristics are listed in Table 1.

**Table 1:** Clinical characteristics, drug resistance genes and virulence-associated genes of 44 CRKP isolates

Isolate no.	Age (yr)	Date of specimen collection (yr/mo/day)	Isolation site(s)	Ward	Underlying disease	Treatment	Outcome	Resistance genes	Virulence genes	MLST
JmsCRE01	45–50	2015/3./11	Sputum	ICU	Brain and abdominal injury, pneumonia	Cefmenoxime, ETP, IMP, SCF, LVX	Recovered	*KPC-2*, *SHV*, *TEM*, *CTX-M-15*	*uge*, *mrkD*, *fimH*, *kpn*	76
JmsCRE02	55–60	2016/12/29	Sputum	Neurosurgery	Brain injury, bacteremia, pneumonia	TZP, CRO, LZD, LVX	Recovered	*KPC-2*, *SHV*, *TEM*, *CTX-M-15*	*uge*, *mrkD*, *fimH*, *kpn*	76
JmsCRE03	75–80	2016/11/21	Sputum	ICU	Lung cancer, cerebral hemorrhage, pneumonia	TZP, IMP, SCF, LVX	Died	*KPC-2*, *SHV*, *TEM*, *CTX-M-15*	*uge*, *mrkD*, *fimH*, *kpn*	76
JmsCRE04	55–60	2016/6/18	Sputum	Neurosurgery	Cerebral hemorrhage, pneumonia	TZP, LVX	Recovered	*KPC-2*, *SHV*, *TEM*, *CTX-M-15*	*uge*, *mrkD*, *fimH*, *kpn*	76
JmsCRE05	65–70	2016/8/2	Sputum	Neurosurgery	Brain injury, cerebral hemorrhage, pneumonia	TZP, LVX, Cefoselis	Recovered	*KPC-2*, *SHV*, *TEM*, *CTX-M-15*	*uge*, *mrkD*, *fimH*, *kpn*	76
JmsCRE06	65–70	2017/1/13	Sputum	Hematology	Cerebral hemorrhage, pneumonia, hypertension	TZP	Died	*KPC-2*, *SHV*	*uge*, *mrkD*, *fimH*, *kpn*	323
JmsCRE07	15–20	2016/8/22	Sputum	Emergency department	Brain injury, hemorrhagic shock, pleural effusion, pneumonia	Cefoperazone/tazobactam, IMP	Recovered	*KPC-2*, *SHV*, *TEM*, *CTX-M-15*	*uge*, *mrkD*, *fimH*, *kpn*	76
JmsCRE08	70–75	2016/11/9	Sputum	Neurosurgery	Cerebral hemorrhage, hypertension, pneumonia, pleural effusion	TZP, CTT, MXF	Recovered	*KPC-2*, *SHV*, *TEM*, *CTX-M-15*	*uge*, *mrkD*, *fimH*, *kpn*	76
JmsCRE09	85–90	2016/8/22	Sputum	ICU	Intestinal obstruction, liver abscess, lung space, pneumonia	Cefoperazone/tazobactam, IMP	Died	*KPC-2*, *SHV*, *TEM*, *CTX-M-15*	*uge*, *mrkD*, *fimH*, *kpn*	76
JmsCRE10	75–80	2016/11/2	Sputum	ICU	Cerebral hemorrhage, pneumonia	–	Recovered	*KPC-2*, *SHV*, *TEM*, *CTX-M-15*	*uge*, *mrkD*, *fimH*, *kpn*	76
JmsCRE11	60–65	2016/11/2	Sputum	Neurosurgery	Cerebral hemorrhage, pneumonia, hypertension	TZP, SCF	Recovered	*KPC-2*, *SHV*, *TEM*, *CTX-M-15*	*uge*, *mrkD*, *fimH*, *kpn*	76
JmsCRE12	75–80	2016/8/29	Sputum	ICU	Cerebral infarction, pericardial effusion, pneumonia	Cefoperazone/tazobactam, VAN, LVX	Died	*KPC-2*, *SHV*, *TEM*, *CTX-M-15*	*uge*, *mrkD*, *fimH*, *kpn*	76
JmsCRE14	60–65	2016/11/14	Sputum	Neurosurgery	Cerebral hemorrhage	TZ, LVX	Recovered	*KPC-2*, *SHV*, *TEM*, *CTX-M-15*	*uge*, *mrkD*, *fimH*, *kpn*	76
JmsCRE15	30–35	2016/11/9	Sputum	Orthopedics	Cervical fracture, pneumonia	CLI, TZP, IMP, SCF, LVX	Recovered	*KPC-2*, *SHV*, *TEM*, *CTX-M-15*	*uge*, *mrkD*, *fimH*, *kpn*	76
JmsCRE16	55–60	2016/6/8	Sputum	ICU	Cerebral hemorrhage, pleural effusion, pneumonia	CTT, LVX	Recovered	*KPC-2*, *SHV*, *TEM*, *CTX-M-15*	*uge*, *mrkD*, *fimH*, *kpn*	76
JmsCRE17	80–85	2016/9/8	Sputum	ICU	Infectious shock, pneumonia	TZP, IMP, MXF	Died	*KPC-2*, *SHV*, *CTX-M-15*	*uge*, *mrkD*, *fimH*, *kpn*	76
JmsCRE18	40–45	2016/4/11	Sputum	ICU	Brain palsy, brain injury	TZP, LVX	Died	*KPC-2*, *SHV*, *CTX-M-15*	*uge*, *mrkD*, *fimH*, *kpn*	76
JmsCRE20	55–60	2016/5/10	Sputum	Neurosurgery	Aneurysm, cerebral hemorrhage	TZP, Ceftezole	Died	*KPC-2*, *SHV*, *CTX-M-15*	*uge*, *mrkD*, *fimH*, *kpn*	76
JmsCRE22	55–60	2017/2/5	Sputum	Emergency department	Gastric cancer, pneumonia	CMZ, TZP, SCF, MXF	Died	*IMP-4*, *SHV*, *TEM*	*uge*, *mrkD*, *fimH*, *kpn*	896
JmsCRE23	55–60	2016/8/22	Blood	ICU	Brain injury, cerebral hemorrhage, peritoneal effusion,	TZP, SCF, IMP	NR	*KPC-2*, *SHV*, *TEM*, *CTX-M-15*	*uge*, *mrkD*, *fimH*, *kpn*	76
JmsCRE24	65–70	2016/7/27	Blood	Neurosurgery	Cerebral hemorrhage, bacteremia, pneumonia, diabetes	CMZ, TZP, AMK, SCF	Recovered	*KPC-2*, *SHV*, *TEM*, *CTX-M-15*	*uge*, *mrkD*, *fimH*, *kpn*	76
JmsCRE28	25–30	2016/10/25	Blood	ICU	Abdominal closure injury, spleen rupture, peritoneal effusion	TZP, IMP	Died	*KPC-2*, *SHV*, *TEM*, *CTX-M-15*	*uge*, *mrkD*, *fimH*, *kpn*	76
JmsCRE29	40–45	2016/8/12	Sputum	Neurosurgery	Cerebral hemorrhage, pneumonia	CMZ, TZP	Recovered	*KPC-2*, *SHV*, *TEM*, *CTX-M-15*	*uge*, *mrkD*, *fimH*, *kpn*	76
JmsCRE30	65–70	2016/7/28	Sputum	ICU	Hydronephrosis, bacteremia, pneumonia	LVX, MXF, IMP, SCF, MSU	Recovered	*KPC-2*, *SHV*, *TEM*, *CTX-M-15*	*uge*, *mrkD*, *fimH*, *kpn*	76
JmsCRE31	55–60	2016/5/27	Sputum	Neurosurgery	Brain abscess, pneumonia	Cefoselis, LVX	Recovered	*KPC-2*, *SHV*, *TEM*, *CTX-M-15*	*uge*	76
JmsCRE32	35–40	2015/10/30	Sputum	Neurosurgery	Brain injury	TZP, LVX	Recovered	*KPC-2*, *SHV*, *TEM*, *CTX-M-15*	*uge*, *mrkD*, *fimH*, *kpn*	76
JmsCRE34	60–65	2016/7/14	Sputum	ICU	Renal failure, uremia, cerebral infarction, pneumonia, pleural effusion	TZP	Recovered	*KPC-2*, *SHV*, *TEM*, *CTX-M-15*	*uge*, *mrkD*, *fimH*, *kpn*	76
JmsCRE35	70–75	2016/7/22	Sputum	Neurosurgery	Cerebral hemorrhage, cerebral infarction, pneumonia	TZP, MXF	Recovered	*KPC-2*, *SHV*, *TEM*, *CTX-M-15*	*uge*, *mrkD*, *fimH*, *kpn*	76
JmsCRE36	70–75	2016/8/28	Wound	ICU	Lower extremity crush sleeve, femoral shaft fracture	TZP, SCF, LVX	Recovered	*KPC-2*, *SHV*, *TEM*, *CTX-M-15*	*uge*, *mrkD*, *fimH*, *kpn*	76
JmsCRE37	55–60	2015/12/23	Sputum	Hematology	Myelodysplastic syndrome, bacteremia	MEM, TZP	Died	*IMP-4*, *SHV*, *TEM*, *CTX-M-15*	*uge*, *mrkD*, *fimH*, *kpn*	2964
JmsCRE39	70–75	2016/11/30	Sputum	Neurosurgery	Cerebral infarction, coronary heart disease, pneumonia, Intracranial infection	TZP, FOX	Recovered	*KPC-2*, *SHV*, *TEM*, *CTX-M-15*	*uge*, *mrkD*, *fimH*, *kpn*	76
JmsCRE44	65–70	2016/4/29	Sputum	Neurosurgery	Intracranial occupying lesions, pneumonia	CFZ, TZP	Died	*KPC-2*, *SHV*, *TEM*, *CTX-M-15*	*uge*, *mrkD*, *fimH*, *kpn*	76
JmsCRE47	55–60	2016/9/30	Blood	Hematology	Aplastic anemia, bacteremia	FOX, Cefoperazone/tazobactam, MXF, MEM	Recovered	*KPC-2*, *SHV*	*uge*, *mrkD*, *fimH*, *kpn*	11
JmsCRE48	75–80	2017/4/28	Sputum	Neurosurgery	Brain palsy, Cerebral hemorrhage, pneumonia	Ceftezole, TZP, MXF	Died	*KPC-2*, *SHV*, *TEM*, *CTX-M-15*	*uge*, *mrkD*, *fimH*, *kpn*	76
JmsCRE49	55–60	2017/6/7	Blood	Cardiac surgery	Brain injury, cerebral hemorrhage, pneumonia, bacteremia	TZP, SCF, Etimicin, FOF, IMP, LVX, AMK	Recovered	*KPC-2*, *SHV*, *TEM*, *CTX-M-15*	*uge*, *mrkD*, *fimH*, *kpn*	76
JmsCRE50	75–80	2017/4/14	Sputum	Neurosurgery	Cerebral infarction, pneumonia, hypertension	TZP, FEP, LVX, IMP, SCF, MXF	Recovered	*KPC-2*, *TEM*, *CTX-M-15*	*uge*, *mrkD*, *fimH*, *kpn*	76
JmsCRE52	45–50	2016/10/6	Sputum	ICU	Brain injury, intracranial infection	ATM, CRO, Cefoselis, LZD	Recovered	*KPC-2*, *TEM*, *CTX-M-15*	*uge*, *mrkD*, *fimH*, *kpn*	76
JmsCRE54	65–70	2016/4/6	Sputum	Neurosurgery	Cerebral infarction, urinary tract infection, pneumonia	TZP, MXF	Recovered	*KPC-2*, *SHV*, *TEM*, *CTX-M-15*	*uge*, *mrkD*, *fimH*, *kpn*	76
JmsCRE55	55–60	2017/4/7	Sputum	ICU	Cerebral hemorrhage, pneumonia	TZP, MEM	NR	*KPC-2*, *SHV*, *TEM*, *CTX-M-15*	*uge*, *mrkD*, *fimH*, *kpn*	76
JmsCRE56	60–65	2017/9/3	Sputum	Neurosurgery	Cerebral hemorrhage, pneumonia, bronchiectasis	TZP	Recovered	*SHV*, *TEM*, *CTX-M-15*	*uge*, *mrkD*, *fimH*, *kpn*	76
JmsCRE57	70–75	2017/11/8	Sputum	ICU	Subarachnoid hemorrhage, cerebral aneurysm, hypostatic pneumonia, hypertension, arrhythmia	Cefoselis, CRO, FOF, Etimicin	Recovered	*KPC-2*, *SHV*, *DHA*, *TEM*, *CTX-M-15*	*uge*, *mrkD*, *fimH*, *kpn*, *aero*, *rmpA*	375
JmsCRE58	40–45	2017/10/14	Sputum	ICU	Brain injury, subarachnoid hemorrhage, skull fracture, chest closure injury, rib fracture	TZP, LVX, Etimicin, VRC	Recovered	*IMP-4*, *SHV*, *TEM*	*uge*, *mrkD*, *fimH*, *kpn*	76
JmsCRE59	55–60	2018/1/16	Sputum	Emergency department	Cerebral infarction, pneumonia, hypertension	CMZ, Etimicin	Recovered	*NDM*, *TEM*, *CTX-M-15*	*uge*, *mrkD*, *fimH*, *kpn*	530
JmsCRE62	35–40	2017/12/13	Sputum	Emergency department	Diabetes ketoacidosis, ion disorder, urinary tract infection, pneumonia, hypoproteinemia, anemia	Cefoperazone/tazobactam, Etimicin	Recovered	*KPC-2*, *SHV*, *DHA*, *TEM*, *CTX-M-15*	*uge*, *mrkD*, *fimH*, *kpn*, *alls*	3335

Note: *ICU* Intensive Care Unit, *ETP* ertapenem, *IMP* imipenem, *SCF* cefoperazone/sulbactam, *LVX* levofloxacin, *TZP* piperacillin/tazobactam, *CRO* ceftriaxone, *LZD* linezolid, *CTT* cefotetan, *MXF* moxifloxacin, *VAN* vancomycin, *CLI* clindamycin, *CMZ* cefmetazole, *AMK* amikacin, *MSU* mezlocillin/sulbactam, *CFZ* cefazolin, *FOX* cefoxitin, *MEM* meropenem, *FOF* fosfomycin, *FEP* cefepime, *ATM* aztreonam, *VRC* voriconazole; −,unmedicated, *NR* no record, *MLST* multilocus sequence typing. Resistance and virulence genes were amplified by PCR.

### Molecular characteristics of CRKP isolates

**Fig. 1:**
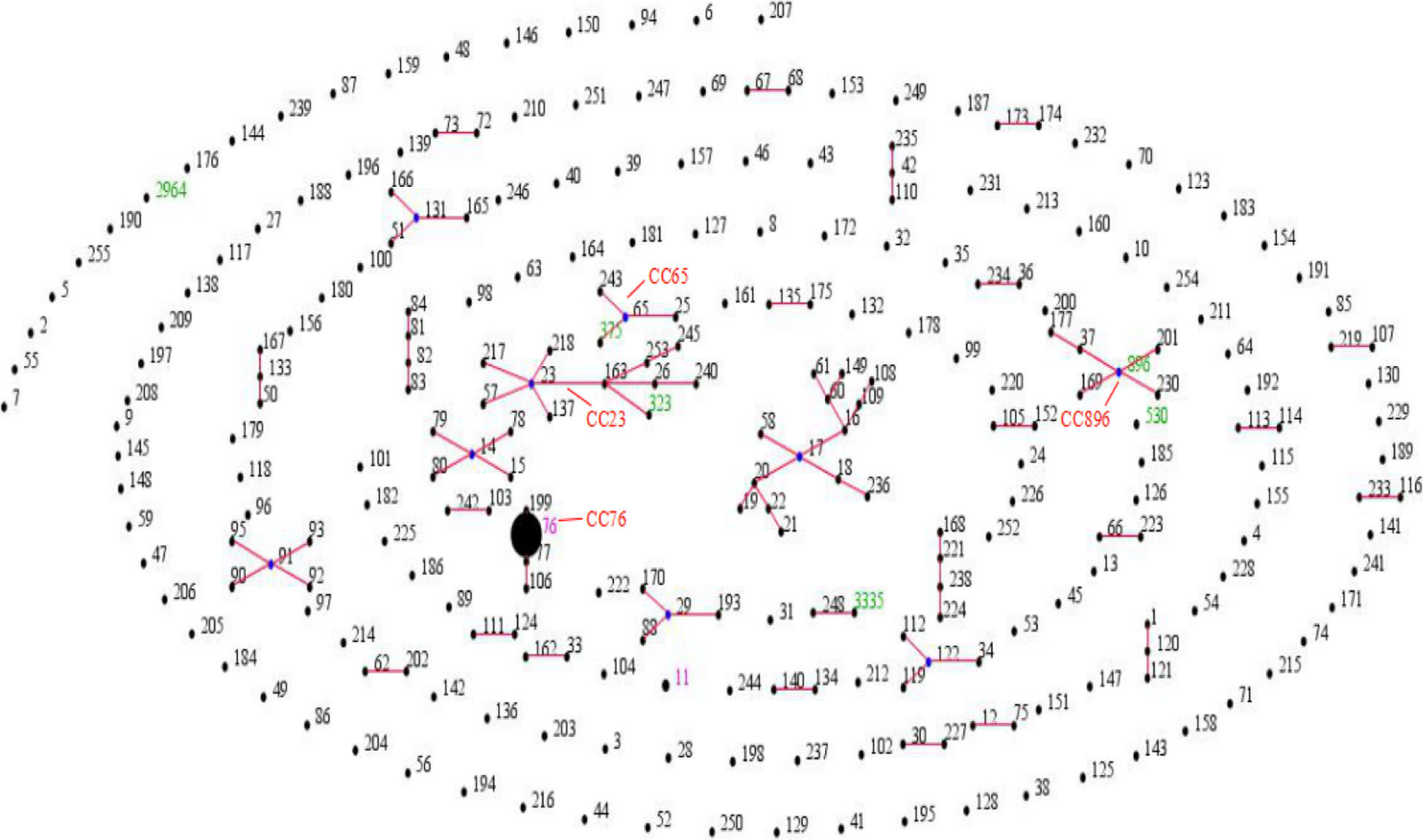
Population snapshot of *K. pneumoniae* by eBURST*.* Note: The STs in this study were compared with the STs in *K. pneumoniae*. Four clonal complexes, namely, CC76, CC65, CC23 and CC896 were identified in the MLST database. Each dot represents one ST, and the size of each dot indicates the number in both databases. The blue dots indicate that the primary founders are positioned centrally; they are connected to the subgroup founders. Black STs correspond to the *K. pneumoniae* MLST database. Green STs correspond to our data. Purple STs correspond to both databases

**Fig. 2:**
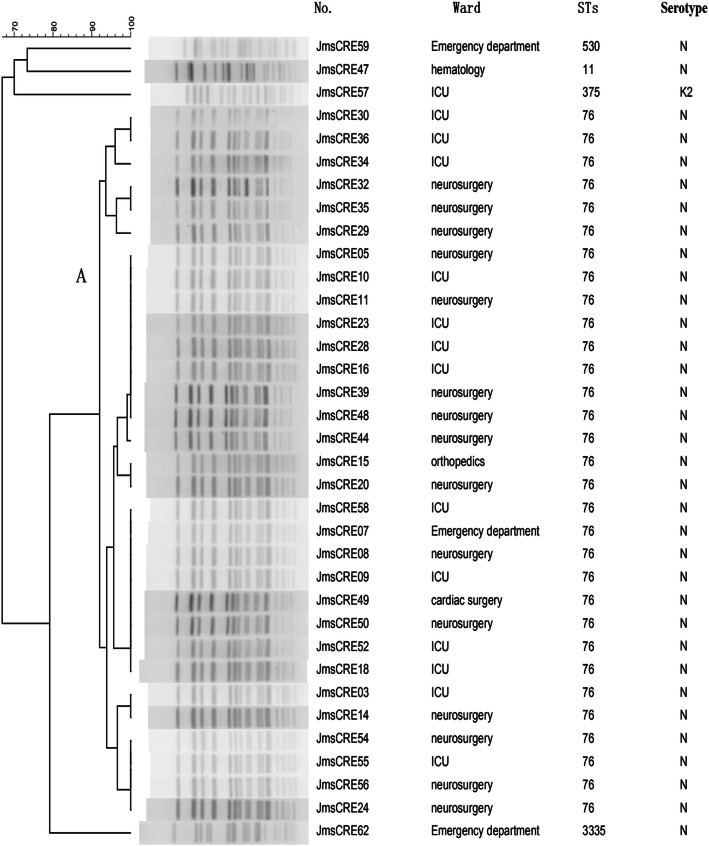
Dendrogram of pulse-field gel electrophoresis developed using BioNumerics Software for 35 CRKP isolates. Note: Clusters were defined as DNA patterns sharing ≥85% similarity. Most of the isolates had a clonal relationship. No., number of isolates; ST: sequence type

Eight STs were identified among 44 CRKP isolates, which included 37 isolates for ST76 and one isolate each for ST11, ST323, ST896, ST2964, ST375, ST530 and ST3335. ST76 (81.8%), the most prevalent ST, belonged to CC76. One carbapenem-resistant hypervirulent *K. pneumoniae* isolate belonged to ST375 (CC65), whereas another isolate, ST3335, was a novel ST. ST323 and ST896 belonged to CC23 and CC896, respectively. There was no clonal complex correlation between STs (Fig. 1). PFGE showed one cluster; it was calculated by the unweighted pair group method with arithmetic mean (UPGMA) using a dice coefficient (Fig. 2). Cluster A had 31 isolates of ST76 (88.57%), which represented the largest group of STs. Within this group, each isolate had a similar PFGE pattern that exceeded SAB 0.9, thus indicating that most of the isolates shared a clonal relationship. ST3335 was similar to cluster A with SAB 0.8. ST530, ST11 and ST375 showed different PFGE patterns with SAB 0.71, suggesting that they had a polyclonal origin.

### Genetic characterization of CRKP isolates

We previously reported the PCR amplification of resistance genes [11]. Each isolate in this study expressed resistance genes (Table 1). The prevalent *bla*_*KPC-2*_ gene was detected in 41 isolates (93.2%), whereas *bla*_IMP-4_ and *bla*_NDM_ genes were detected in only two isolates (4.5% each). The prevalent β-lactamase genes *bla*_SHV_, *bla*_CTX-M_ and *bla*_TEM_ were mostly expressed by CRKP isolates at ratios of 95.5% (42/44), 90.9% (40/44) and 90.9% (40/44), respectively. Two isolates expressed *bla*_*DHA*_ (4.5% each). Each CRKP isolate in this study expressed at least two resistance genes, whereas 68.2% CRKP strains co-expressed *bla*_KPC-2_, *bla*_CTX-M-15_, *bla*_SHV_ and *bla*_TEM_ genes.

### String test, capsular serotyping and virulence-associated genes among CRKP isolates

Only one (JmsCRE57) out of 44 CRKP isolates (2.3%) exhibited the hypermucoviscous phenotype during the string test and capsular serotyping. The remaining 43 CRKP isolates were not successfully serotyped. The virulence-associated genes detected by PCR for each isolate are listed in Table 1. The carbapenem-resistant hypervirulent *K. pneumoniae* K2 serotype expressed several virulence-associated genes, including *uge*, *mrkD*, *fimH*, *kpn*, *aerobactin* and *rmpA*. Each CRKP isolate in this study expressed the *uge* gene. Most CRKP isolates expressed *fimH* (97.7%), *mrkD* (97.7%) and *kpn* (97.7%). Only JmsCRE62 expressed the *alls* gene, and the detection rate was 2.4% for *aerobactin*, *rmpA* and *alls* genes. None of the isolates expressed *magA*, *kfu*, *iroNB* and *wcaG* genes.

### Biofilm formation

**Fig. 3:**
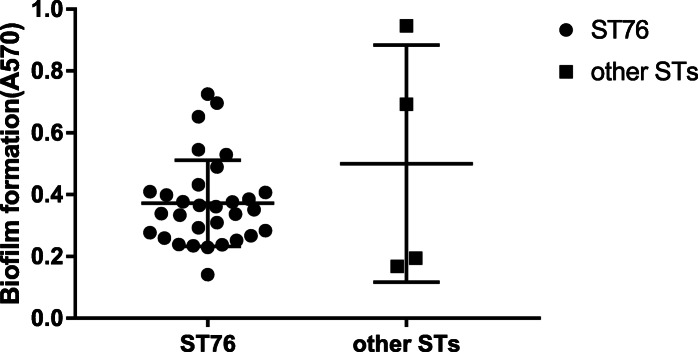
Biofilm formation in 35 CRKP isolates. Note: The circles indicate ST76; the squares indicate other STs. In the other STs group, biofilm production was strongest in ST3335 JmsCRE62, followed by ST375 JmsCRE57, ST11 JmsCRE47 and ST530 JmsCRE59. Isolates belonging to the ST76 lineage formed significantly less biofilm than the other group (median A570 of 0.35 vs. 0.44, respectively)

Biofilm formation was observed in 35 CRKP isolates (Fig. 3). The highest biofilm producer was ST3335 isolate JmsCRE62 (0.95), which did not exhibit the capsule serotype and the hypermucoviscous phenotype. The second highest biofilm producer was ST76 isolate JmsCRE54 (0.73), whereas ST375 isolate JmsCRE57 (0.69) was the third highest. Approximately 14.3% (5/35) of the isolates were classified as strong-producers, 17.1% (6/35) as moderate-producers and 68.6% (24/35) as weak-producers. Compared with the other STs (median A570 of 0.44), ST76 was the low biofilm producer (median A570 of 0.35).

### Serum complement-mediated killing resistance

**Fig. 4:**
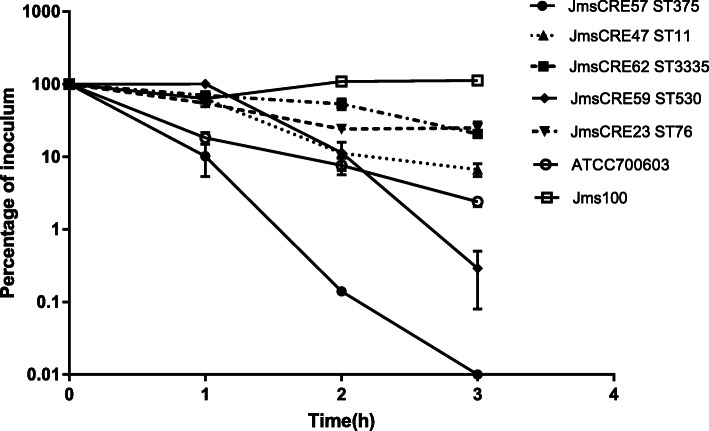
Serum complement-mediated killing of selected CRKP isolates. Note: In vitro growth/survival of *K. pneumoniae* isolates JmsCRE62, JmsCRE47, JmsCRE23, ATCC 700603 and Jms100 in 90% human serum. The JmsCRE57 isolate died. The values are presented as means ± SEM of triplicates

The results of the serum complement-mediated killing assay are shown in Fig. 4. Jms100, which was isolated from a liver abscess and exhibited a hypermucoviscous phenotype, was sensitive to all antimicrobials, except ampicillin. ST375 isolate JmsCRE57, ST3335 isolate JmsCRE62, ST11 isolate JmsCRE47, ST76 isolate JmsCRE23 and ATCC 700603 were all sensitive to serum complement-mediated killing (grade 2, 0, 20.72, 6.68, 25.03 and 2.4%, respectively). JmsCRE57, which was highly sensitive to serum complement-mediated killing, died within 3 h. ST530 isolate JmsCRE59 was moderately sensitive to serum complement-mediated killing (grade 3, 0.29%). The growth rate of Jms100 was the highest; however, which expressed serum resistance (grade 6, 112.2%) and successfully avoided the complement-mediated serum killing in vivo.

### Genome sequencing and analysis

**Fig. 5:**
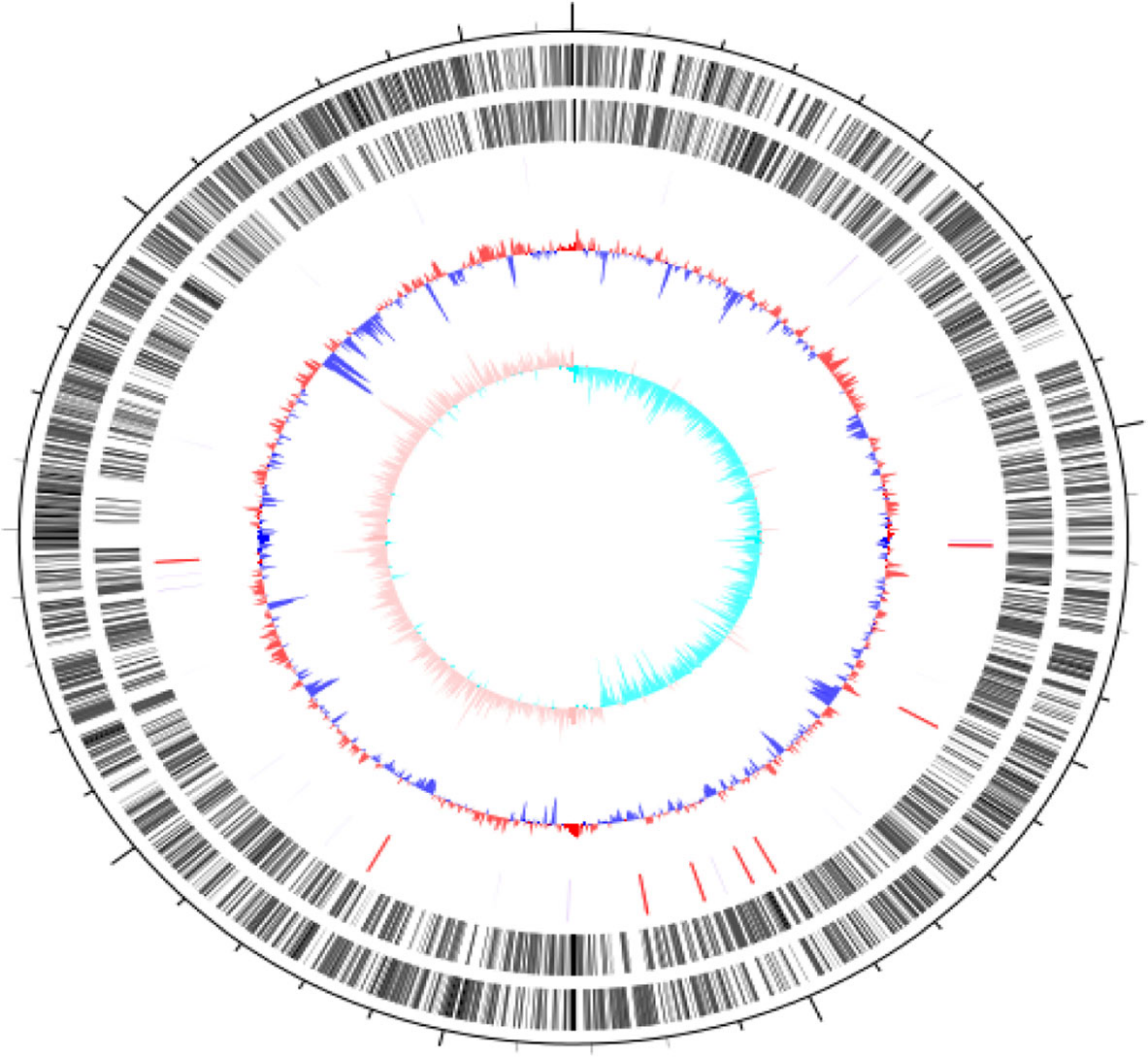
Schematic circular genome of JmsCRE57. Note: The second and third circles from the outside to the inside represent the CDS on the positive and negative chains. The fourth circle represents rRNA and tRNA. The fifth circle represents the GC content, and the outer red portion indicates that the GC content in this region was higher than the average GC content of the whole genome. The innermost circle represents the GCskew value

**Fig. 6:**
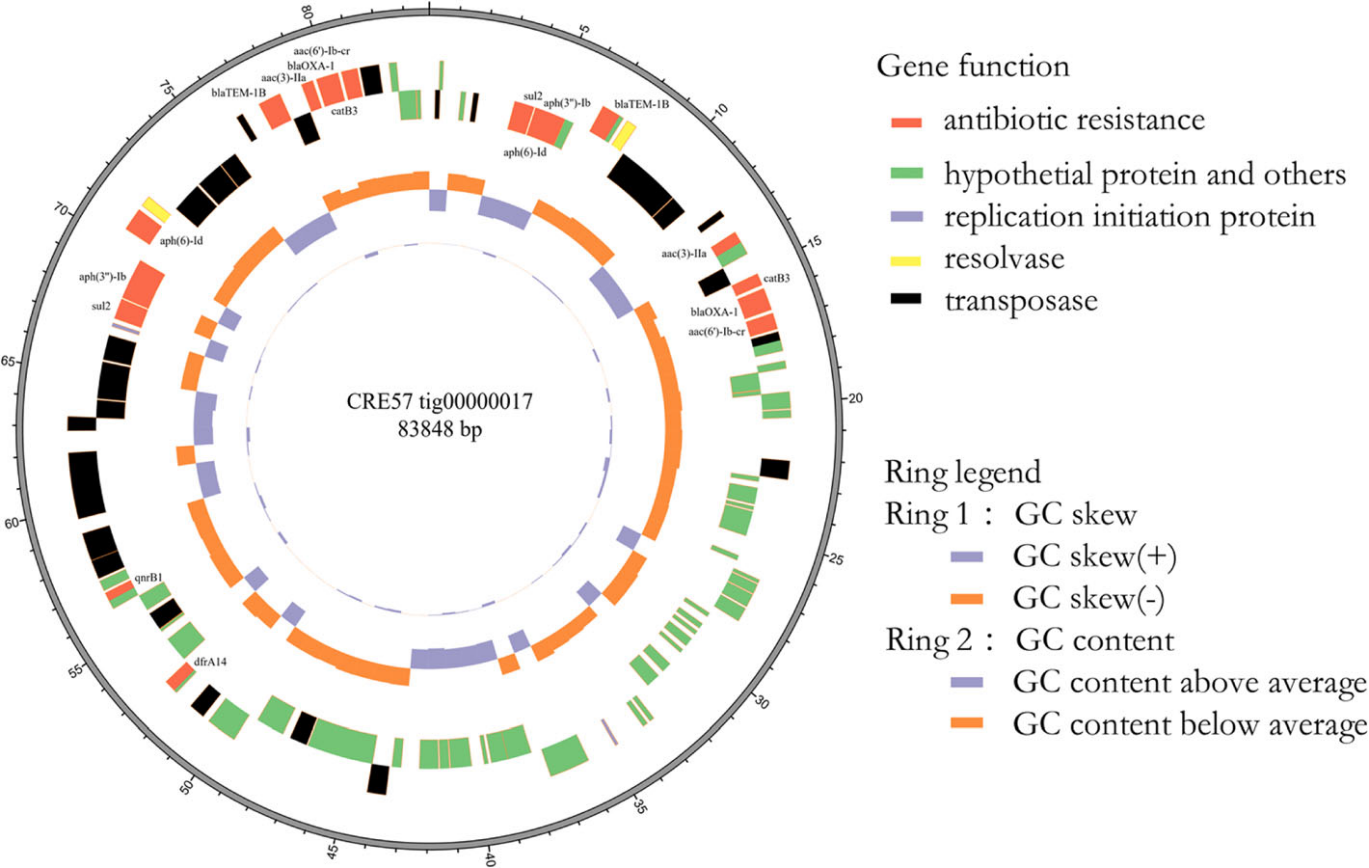
Circular map of plasmid tig00000017 of JmsCRE57. Note: The one circle from the inside to the outside represents the GCskew value. The second circle represents the GC content, and the outer red portion indicates that the GC content in this region was lower than the average GC content of the whole genome. The third circle of each color represents the corresponding gene function. Antimicrobial resistance genes are indicated

**Fig. 7:**
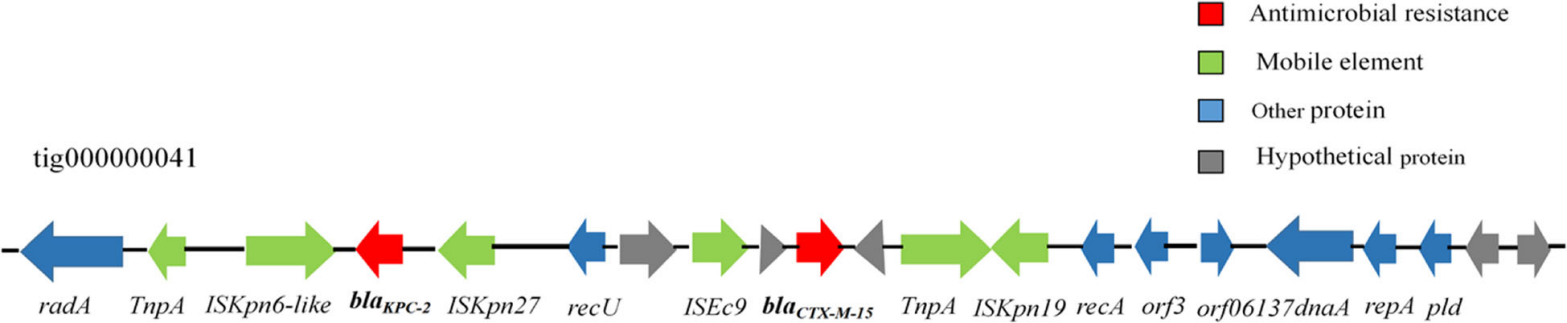
Schematic representation of the genetic environment of *bla*_*KPC-2*_ genes on tig00000041 plasmid. Note: The gene name is shown next to the corresponding arrow or rod

The sequencing of JmsCRE57 revealed a total of 7,457,750 (1,098,918,433 bp) paired-end reads that were generated with the Illumina HiSeq 2000 System and 62,801 (417,488,124 bp) raw reads that were produced with the PacBio System. Our analysis showed that the JmsCRE57 genome consisted of a circular chromosome of 4,649,643 bp and three antimicrobial resistance plasmids of tig00000041 (121,129 bp), tig00000017 (83,848 bp) and tig00000012 (688,226 bp), and a virulent tig00000014 plasmid (199,142 bp). The chromosome features of JmsCRE57 are summarized in Fig. 5. Aminoglycoside resistance genes *aph(3″)-Ib* and *aph(6)-Id*, the quinolone resistance gene *qnrB1*, fluoroquinolone and aminoglycoside resistance genes *aac(3)-IIa* and *aac(6′)-Ib-cr*, β-lactamase resistance genes *bla*_*OXA-1*_ and *bla*_*TEM-1B*_, the phenicol resistance gene *catB4*, the sulphonamide resistance gene *sul2* and the trimethoprim resistance gene *dfrA14* were also expressed by the tig00000017 plasmid. The tig00000017 plasmid resistance genes are shown in Fig. 6. The plasmid carrying the carbapenem resistance gene *bla*_*KPC-2*_ and the extended-spectrum β-lactamase gene *bla*_*CTX-M-15*_ on the tig00000041 plasmid belonged to the IncFIB (pQil) incompatibility group. The plasmid carrying the extended-spectrum β-lactamase gene *bla*_*SHV-99*_ on the tig00000012 plasmid. BLASTn analysis revealed that the tig00000041 plasmid, with a 48% query coverage, was 99% similar to the pKPHS2 plasmid (GenBank accession number CP003224.1), which was isolated from a patient in Shanghai. A schematic representation of the genetic environment of the *bla*_*KPC-2*_ and *bla*_*CTX-M-15*_ genes on the tig00000041 plasmid is shown in Fig. 7.

**Fig. 8:**
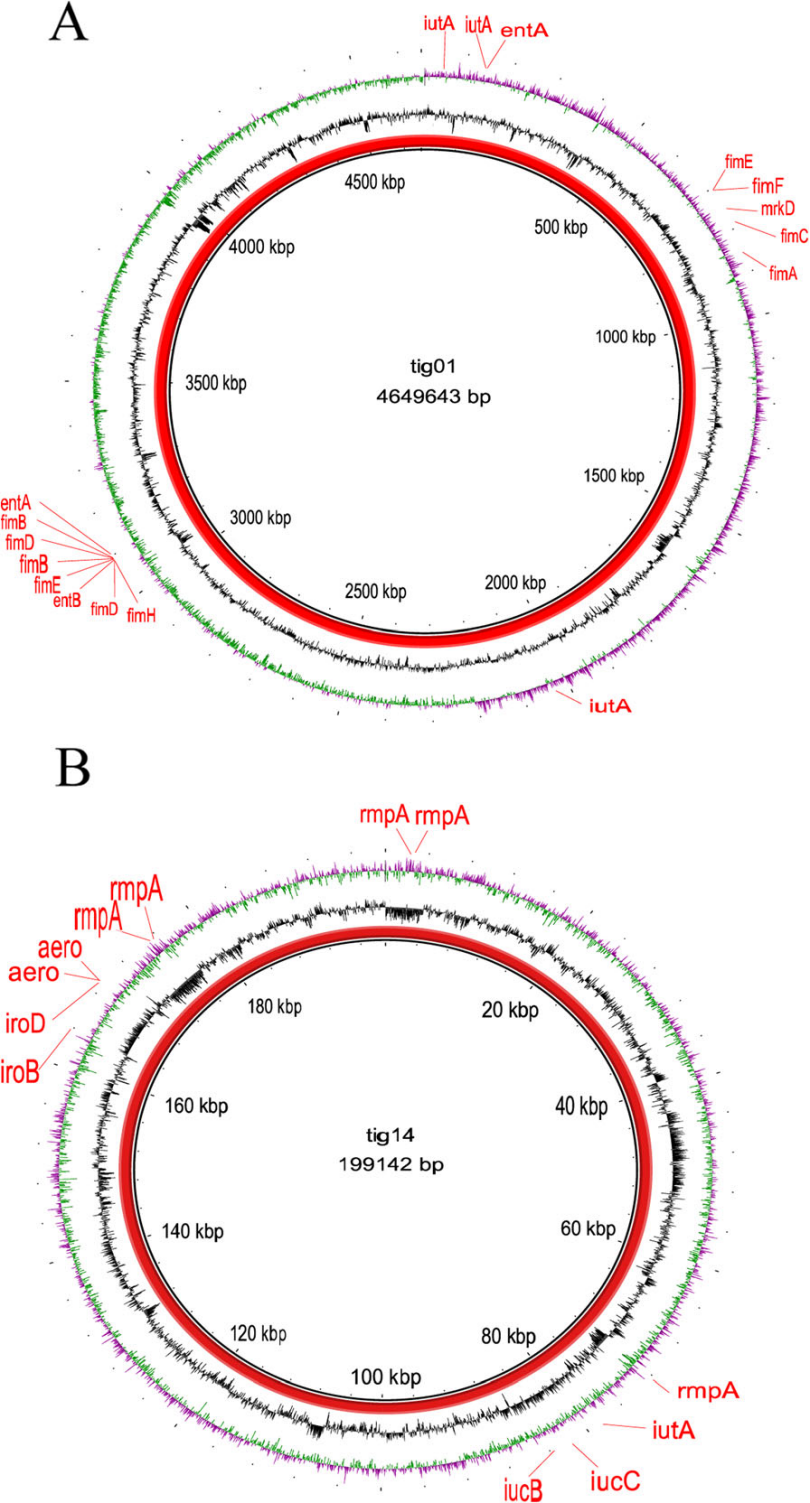
Gene map of virulence plasmid and chromosome harboured by ST375 *K. pneumoniae.* Note: **a** The circular map of virulence gene of chromosome. **b** The circular map of virulence plasmid tig00000014

JmsCRE57-associated virulence genes mainly included the capsular polysaccharide gene *rmpA*; siderophore-associated genes *iucBC*, *iutA*, *iroBD* and *aerobactin* present on the tig00000014 plasmid; fimbrial adhesin genes *fimA-H* and *mrkD* and siderophore-associated genes *iutA* and *entAB* present on the chromosome (Fig. 8).

## Discussion

This retrospective study was conducted on 44 cases presenting with various diseases caused by CRKP from January 2015 to December 2017 at the First Affiliated Hospital of Jiamusi University in Heilongjiang Province, northeast China. The antimicrobial susceptibility of 44 CRKP isolates was previously reported [11]. All strains were resistant to carbapenem; the resistance rate to β-lactamase inhibitor combinations, third-generation cephems and aminoglycosides was 93.18, 100 and 93.18%, respectively. All isolates were sensitive to amikacin, and the resistance rate to levofloxacin was 18.2%. The detection rate of the *bla*_*KPC-2*_ gene, a key enzyme of carbapenem resistance, was 93.2%. The detection rates of the extended-spectrum β-lactamases *bla*_*SHV*_, *bla*_*CTX-M*_ and *bla*_*TEM*_ were 95.46, 86.36 and 90.9%, respectively. Taken collectively, these results support the contention that the resistance of CRKP strains is caused by the expression of multiple resistance genes. Here, ST76 was the predominant clone (81.8%), and the PFGE pattern, which exceeded SAB 0.9, showed that all ST76 isolates shared a clonal relationship. The CRKP isolates investigated in this study mainly concentrated in the neurosurgery unit and ICU, suggesting that there might have been an outbreak of ST76 CRKP that subsequently spread to additional units.

HvKP strains are often identified by a positive string test. However, not all hvKP strains exhibit the hypermucoviscous phenotype, which may lead to the undetection of many hvKPs [14, 18]. *Aerobactin*, a key virulence gene, mediates iron transport in bacteria; it has also been used in the identification of hvKP [19]*.* Here, only one CRKP isolate exhibited a positive string test, and this isolate expressed virulence-associated genes *uge*, *mrkD*, *fimH*, *kpn*, *aerobactin* and *rmpA*. According to the aforementioned criteria, this isolate was identified as a carbapenem-resistant hypervirulent *K. pneumoniae* strain with a ST375 K2 serotype. Interestingly, a previous study reported that ST23, the most prevalent hvKP, strongly correlated with the K1 serotype. However, MLSTs, such as ST65, ST66, ST86, ST374, ST375 and ST380, also associated with the K2 serotype [20]. Our results showed a single locus difference between ST375 and ST65. Likewise, another earlier study reported that ST375 belonged to the K2 serotype and was sensitive to most antimicrobials [21], whereas JmsCRE57 was resistant to most antimicrobials, except amikacin, polymyxin and tigecycline. On the other hand, Guo et al. reported that K2 serotype isolates caused more invasive infections than K1 serotype isolates [1], which is consistent with our findings on the patient with spontaneous subarachnoid hemorrhage. Thus, an understanding of the genetic background and virulence of hvKP strains is crucial.

HvKP strains are characterized by the presence of capsular polysaccharides (K antigen), fimbriae, lipopolysaccharides (O antigen) and siderophores (aerobaction and yersiniabactin) [14]. Here, we investigated 11 virulence-associated genes in 44 CRKP isolates. We found that these isolates expressed *fimH*, *mrkD* and *kpn* genes at a rate of 97.7%, and almost existed in all CRKP strains. The *fimH* gene encodes type 1 fimbrial and the *mrkD* gene encodes type 3 fimbrial, which play critical roles in adhesion to the respiratory tract and urethra, as well as in bacterial infections and biofilm formation. The *mrkD* gene, regardless of whether it is hypervirulent or non-hypervirulent [22], is often detected in cases of ventilator-associated pneumonia caused by *K. pneumoniae*. Here, 83.8% of the isolates were mainly harvested from respiratory tract secretions, accounting for 72.7% of the total number of patients diagnosed with pneumonia. Furthermore, only JmsCRE62 expressed the *alls* gene, which mediates allantoin metabolism and facilitates the development of liver abscesses caused by *K. pneumoniae*. Although a previous study reported a strong correlation between the *alls* gene and the K1 serotype [23], JmsCRE62 was unsuccessfully serotyped in this study.

Biofilm formation inhibits the penetration of drugs, thus increasing antibiotic resistance, which further complicates the clinical treatment of *K. pneumoniae* infections [24]. *K. pneumoniae* strains can also avoid phagocytosis by neutrophils, thus causing refractory and chronic infections. A previous study reported that biofilm formation required the type 3 fimbrial and adhesion factor *mrkD* [25]. Here, the detection rate of *mrkD* was 97.7%, whereas that of strong biofilm was 14.3%, which signifies a significant difference. Biofilm formation also involves different biomolecules, including extracellular polysaccharides, proteins and DNA.

The complement system, an important component of the immune system in humans, promotes the membrane attack and phagocytosis of foreign cells such as bacteria. *K. pneumoniae* produce capsular polysaccharides that make this species of bacteria resistant to serum complement-mediated killing, thus promoting their survival. Although JmsCRE57 was the only strain in this study to produce capsular polysaccharides, it was killed by the complement system, which is different from many carbapenem-resistant hypervirulent *K. pneumoniae* strains that are resistant to serum complement-mediated killing [7, 26, 27]. JmsCRE57 was harvested from a 71-year-old female with hypertension and arrhythmia, who was hospitalized 9 h after suffering from a spontaneous subarachnoid hemorrhage. This patient was previously treated with cefoselis and ceftriaxone, and she was hospitalized in the neurosurgery unit and ICU for 48 days. In addition to being sensitive to amikacin and tigecycline, this strain is also resistant to quinolones, aminoglycosides, macrolides, cephalosporins, β-lactamase inhibitor combinations and carbapenems. During hospitalization, the patient was not treated with antimicrobials, except that she received fosfomycin and etimicin to prevent urinary tract infections due to catheter use. Subsequently, her health improved, and the patient was discharged, suggesting that this strain was sensitive to serum complement-mediated killing. Multi-drug resistant bacteria are generally considered to have higher fitness or less virulence [28]. Gottig suggests that the acquisition of new plasmids and other mobile genetic elements can reduce fitness [29]. The whole genome sequencing results showed that JmsCRE57 mainly contained three antibiotic resistant plasmids and one virulence plasmid, which increased the fitness cost of the strain, rendering it easily killed by the immune system. Further studies are needed on the fitness of carbapenem-resistant hypervirulent *K. pneumoniae* strains.

The tig00000014 virulence plasmid in JmsCRKP57 belonged to the IncHI1B group, similar to the pLVPK (AY378100) virulence plasmid belonging to the IncHI1B/IncFIB group that was collected from *K. pneumoniae* CG43, which was mainly composed of mucoid phenotype genes and siderophore-associated genes. JmsCRKP57 also had *fimA-H*, *mrkD*, *iutA* and *entAB* genes on the chromosome, which might also be typical of ST375 *K. pneumoniae*.

The horizontal transmission of mobile genes, such as plasmids, phages, integration and conjugated elements and insertion elements, is a key factor in the prevalence of *K. pneumoniae* outbreaks [30]. Here, the tig00000041 plasmid expressing both *bla*_*KPC-2*_ and *bla*_*CTX-M-15*_ genes was identified; it was located on the TnpA transposon and found to have insertion elements at both ends. When a transposon is inserted into different plasmid backbones, new KPC-2 and CTX-M plasmid can be formed. This phenomenon might have caused the outbreak at the hospital. Presently, two mechanisms can explain the development of carbapenem-resistant hypervirulent *K. pneumoniae* strains. In the first mechanism, Siu et al. reported successful transfer of a KPC-producing plasmid into a hvKP strain, which no longer only resisted ampicillin and streptomycin but also all β-lactams without losing virulence [31]. In the second mechanism, Gu et al. reported successful transfer of a 170-kbp pLVPK-like virulent plasmid into ST11 CRKP, which formed ST11 CRKP with K1 hypervirulence [10]. A plasmid expressing the *bla*_*CTX-M*_ gene has also been shown to be compatible with various hvKP strains [32]. If large-scale horizontal transmission is possible, hvKP strains can become highly resistant to antimicrobials. Here, 86.36% of CRKP isolates expressed the *bla*_*CTX-M*_ gene, suggesting that this high carrier rate might facilitate horizontal transmission and lead to the formation of a highly resistant hvKP strain. Regardless, both mechanisms can result in a widespread outbreak of carbapenem-resistant hypervirulent *K. pneumoniae* strains; therefore, effective control measures are critical.

## Conclusions

To our best knowledge, we are the first group to report the genetic background and virulence characteristics of the carbapenem-resistant K2 hypervirulent *K. pneumoniae* ST375 isolate in northeast China. This isolate expressed multiple antimicrobial resistance and virulence genes. Furthermore, our study identified an outbreak of KPC-2 CRKP ST76 in a hospital in Heilongjiang Province, northeast China, which was caused by classic *K. pneumoniae* strains; however, both strains expressed adherence virulence genes. The outbreak of CRKP strains and emergence of hypervirulence forces us to promote awareness and to strengthen epidemiological surveillance and infection control measures in our hospital.

## Data Availability

The datasets analysed during the current study are available from the corresponding author on reasonable request.

## Abbreviations

CRKP: Carbapenem-resistant *K. pneumoniae*
MLST: Multilocus sequence typing
PFGE: Pulsed-field gel electrophoresis
hvKP: Hypervirulent *K. pneumoniae*
*rmpA*: Regulate the mucoid phenotype A
*aerobactin*: Siderophore production
KPC: *K. pneumoniae* carbapenemases
NDM: New Delhi metallo-beta-lactamase
MICs: Minimal inhibitory concentrations
ATCC: American Type Culture Collection
STs: Sequence types
CCs: Clonal complexes
SLV: Single locus variant
CFUs: Colony-forming units
LB: Luria-Bertani

## References

[1] Guo Y, Wang S, Zhan L, Jin Y, Duan J, Hao Z, Lv J, Qi X, Chen L, Kreiswirth BN (2017). Microbiological and clinical characteristics of Hypermucoviscous Klebsiella pneumoniae isolates associated with invasive infections in China. Front Cell Infect Microbiol.

[2] Zheng B, Dai Y, Liu Y, Shi W, Dai E, Han Y, Zheng D, Yu Y, Li M (2017). Molecular epidemiology and risk factors of Carbapenem-resistant Klebsiella pneumoniae infections in eastern China. Front Microbiol.

[3] Siu LK, Fung CP, Chang FY, Lee N, Yeh KM, Koh TH, Ip M (2011). Molecular typing and virulence analysis of serotype K1 Klebsiella pneumoniae strains isolated from liver abscess patients and stool samples from noninfectious subjects in Hong Kong, Singapore, and Taiwan. J Clin Microbiol.

[4] Yao B, Xiao X, Wang F, Zhou L, Zhang X, Zhang J (2015). Clinical and molecular characteristics of multi-clone carbapenem-resistant hypervirulent (hypermucoviscous) Klebsiella pneumoniae isolates in a tertiary hospital in Beijing, China. Int J Infect Dis.

[5] Bocanegra-Ibarias P, Garza-Gonzalez E, Morfin-Otero R, Barrios H, Villarreal-Trevino L, Rodriguez-Noriega E, Garza-Ramos U, Petersen-Morfin S, Silva-Sanchez J (2017). Molecular and microbiological report of a hospital outbreak of NDM-1-carrying Enterobacteriaceae in Mexico. PLoS One.

[6] Struve C, Roe CC, Stegger M, Stahlhut SG, Hansen DS, Engelthaler DM, Andersen PS, Driebe EM, Keim P, Krogfelt KA (2015). Mapping the Evolution of Hypervirulent *Klebsiella pneumoniae*. mBio.

[7] Liu Y, Liu PP, Wang LH, Wei DD, Wan LG, Zhang W (2017). Capsular polysaccharide types and virulence-related traits of epidemic KPC-producing Klebsiella pneumoniae isolates in a Chinese University hospital. Microb Drug Resist.

[8] Diago-Navarro E, Chen L, Passet V, Burack S, Ulacia-Hernando A, Kodiyanplakkal RP, Levi MH, Brisse S, Kreiswirth BN, Fries BC (2014). Carbapenem-resistant Klebsiella pneumoniae exhibit variability in capsular polysaccharide and capsule associated virulence traits. J Infect Dis.

[9] Yang Z, Liu W, Cui Q, Niu W, Li H, Zhao X, Wei X, Wang X, Huang S, Dong D (2014). Prevalence and detection of Stenotrophomonas maltophilia carrying metallo-beta-lactamase blaL1 in Beijing, China. Front Microbiol.

[10] Gu D, Dong N, Zheng Z, Lin D, Huang M, Wang L, Chan EW-C, Shu L, Yu J, Zhang R (2018). A fatal outbreak of ST11 carbapenem-resistant hypervirulent Klebsiella pneumoniae in a Chinese hospital: a molecular epidemiological study. Lancet Infect Dis.

[11] Gong X, Zhang J, Su S, Fu Y, Bao M, Wang Y, Zhang X (2018). Molecular characterization and epidemiology of carbapenem non-susceptible Enterobacteriaceae isolated from the eastern region of Heilongjiang Province, China. BMC Infect Dis.

[12] Liang Y, Yin X, Zeng L, Chen S (2017). Clonal replacement of epidemic KPC-producing Klebsiella pneumoniae in a hospital in China. BMC Infect Dis.

[13] Fang CT, Lai SY, Yi WC, Hsueh PR, Liu KL, Chang SC (2007). Klebsiella pneumoniae genotype K1: an emerging pathogen that causes septic ocular or central nervous system complications from pyogenic liver abscess. Clin Infect Dis.

[14] Fu L, Huang M, Zhang X, Yang X, Liu Y, Zhang L, Zhang Z, Wang G, Zhou Y (2018). Frequency of virulence factors in high biofilm formation blaKPC-2 producing Klebsiella pneumoniae strains from hospitals. Microb Pathog.

[15] El Fertas-Aissani R, Messai Y, Alouache S, Bakour R (2013). Virulence profiles and antibiotic susceptibility patterns of Klebsiella pneumoniae strains isolated from different clinical specimens. Pathologie-biologie.

[16] Jian-Li W, Yuan-Yuan S, Shou-Yu G, Fei-Fei D, Jia-Yu Y, Xue-Hua W, Yong-Feng Z, Shi-Jin J, Zhi-Jing X (2017). Serotype and virulence genes of Klebsiella pneumoniae isolated from mink and its pathogenesis in mice and mink. Sci Rep.

[17] Podschun R, Sievers D, Fischer A, Ullmann U (1993). Serotypes, hemagglutinins, siderophore synthesis, and serum resistance of Klebsiella isolates causing human urinary tract infections. J Infect Dis.

[18] Zhang R, Lin D, Chan EW, Gu D, Chen GX, Chen S (2016). Emergence of Carbapenem-resistant serotype K1 Hypervirulent Klebsiella pneumoniae strains in China. Antimicrob Agents Chemother.

[19] Russo TA, Olson R, MacDonald U, Beanan J, Davidson BA (2015). Aerobactin, but not yersiniabactin, salmochelin, or enterobactin, enables the growth/survival of hypervirulent (hypermucoviscous) Klebsiella pneumoniae ex vivo and in vivo. Infect Immun.

[20] Wang X, Xie Y, Li G, Liu J, Li X, Tian L, Sun J, Ou HY, Qu H (2018). Whole-genome-sequencing characterization of bloodstream infection-causing hypervirulent Klebsiella pneumoniae of capsular serotype K2 and ST374. Virulence.

[21] Liao CH, Huang YT, Chang CY, Hsu HS, Hsueh PR (2014). Capsular serotypes and multilocus sequence types of bacteremic Klebsiella pneumoniae isolates associated with different types of infections. Eur J Clin Microbiol Infect Dis.

[22] Yan Q, Zhou M, Zou M, Liu WE (2016). Hypervirulent Klebsiella pneumoniae induced ventilator-associated pneumonia in mechanically ventilated patients in China. Eur J Clin Microbiol Infect Dis.

[23] Yu WL, Ko WC, Cheng KC, Lee CC, Lai CC, Chuang YC (2008). Comparison of prevalence of virulence factors for Klebsiella pneumoniae liver abscesses between isolates with capsular K1/K2 and non-K1/K2 serotypes. Diagn Microbiol Infect Dis.

[24] Vuotto C, Longo F, Balice MP, Donelli G, Varaldo PE (2014). Antibiotic Resistance Related to Biofilm Formation in *Klebsiella pneumoniae*. Pathogens (Basel, Switzerland).

[25] Stahlhut SG, Chattopadhyay S, Kisiela DI, Hvidtfeldt K, Clegg S, Struve C, Sokurenko EV, Krogfelt KA (2013). Structural and population characterization of MrkD, the adhesive subunit of type 3 fimbriae. J Bacteriol.

[26] Mei YF, Liu PP, Wan LG, Liu Y, Wang LH, Wei DD, Deng Q, Cao XW (2017). Virulence and genomic feature of a virulent Klebsiella pneumoniae sequence type 14 strain of serotype K2 harboring blaNDM-5 in China. Front Microbiol.

[27] Chiang TT, Yang YS, Yeh KM, Chiu SK, Wang NC, Lin TY, Huang LY, Chang FY, Siu LK, Lin JC (2016). Quantification and comparison of virulence and characteristics of different variants of carbapenemase-producing Klebsiella pneumoniae clinical isolates from Taiwan and the United States. J Microbiol Immunol Infection.

[28] Hennequin C, Robin F (2016). Correlation between antimicrobial resistance and virulence in Klebsiella pneumoniae. Eur J Clin Microbiol Infect Dis.

[29] Gottig S, Riedel-Christ S, Saleh A, Kempf VA, Hamprecht A (2016). Impact of blaNDM-1 on fitness and pathogenicity of Escherichia coli and Klebsiella pneumoniae. Int J Antimicrob Agents.

[30] Chen L, Mathema B, Chavda KD, DeLeo FR, Bonomo RA, Kreiswirth BN (2014). Carbapenemase-producing Klebsiella pneumoniae: molecular and genetic decoding. Trends Microbiol.

[31] Siu LK, Huang DB, Chiang T (2014). Plasmid transferability of KPC into a virulent K2 serotype Klebsiella pneumoniae. BMC Infect Dis.

[32] Zhang Y, Zhao C, Wang Q, Wang X, Chen H, Li H, Zhang F, Li S, Wang R, Wang H (2016). High prevalence of Hypervirulent Klebsiella pneumoniae infection in China: geographic distribution, clinical characteristics, and antimicrobial resistance. Antimicrob Agents Chemother.

